# Crystal structure of *N*-butyl-2,3-bis­(di­cyclo­hexyl­amino)­cyclo­propeniminium chloride benzene monosolvate

**DOI:** 10.1107/S2056989022008076

**Published:** 2022-08-23

**Authors:** Gaby M. Muñoz Sánchez, Michael J. Zdilla

**Affiliations:** a Department of Chemistry, Temple University, 1901 N. 13th Street, Philadelphia, PA 19122, USA; Universidad Nacional Autónoma de México, México

**Keywords:** crystal structure, cyclo­propene, superbases, aromaticity

## Abstract

*N*-Butyl-2,3-bis­(di­cyclo­hexyl­amino)­cyclo­propenimine (**1**) crystallizes from benzene and hexa­nes in the presence of HCl as a mono­benzene solvate of the hydro­chloride salt, [**1**H]Cl·C_6_H_6_ or C_31_H_54_N_3_^+^·Cl^−^·C_6_H_6_, in the *P*2_1_/*n* space group. The protonation of **1** results in the generation of an aromatic structure based upon the delocalization of the cyclo­propene double bond around the cyclo­propene ring, giving three inter­mediate C—C bond lengths of ∼1.41 Å, and the delocalization of the imine-type C—N double bond, giving three inter­mediate C—N bond lengths of ∼1.32 Å. Ion–ion and ion–benzene packing inter­actions are described and illustrated.

## Abstract 2

The structure of the acid chloride salt of the superbase *N*-butyl-2,3-bis­(di­cyclo­hexyl­amino)­cyclo­propenimine is reported.

## 1. Chemical context

Penta­substituted di­amino­propenimines are a relatively new class of superbases that operate *via* the establishment of a stable aromatic electronic delocalization upon protonation. Originally reported as four-electron Lewis donors (Bruns *et al.*, 2010 ▸), a more recently exploited application for the use of penta­substituion is that of a superbase, with one of the six nitro­gen coordination sites available for protonation, making these mol­ecules facile initiators of stereoselective Michael (Bandar & Lambert, 2012 ▸) and Mannich reactions (Bandar & Lambert, 2013 ▸), hydro­aminations (Mirabdolbaghi & Dudding, 2015 ▸), and ring-opening polymerization (Stukenbroeker *et al.*, 2015 ▸; Xu *et al.*, 2018 ▸). A number of examples of acid salts of these species have been structurally characterized, permitting direct observation of the aromatized cyclo­propeniminium structures (Stukenbroeker *et al.*, 2015 ▸; Bruns *et al.*, 2010 ▸; Bandar *et al.*, 2015 ▸; Belding & Dudding, 2014 ▸; Guest *et al.*, 2020 ▸; Kozma *et al.*, 2015 ▸; Belding *et al.*, 2016 ▸; Bandar & Lambert, 2012 ▸, 2013 ▸; Mirabdolbaghi & Dudding, 2015 ▸). Examples of free-base penta­substituted di­amino­propenimines are uncommon, and these are typically only obtained with aromatic substituents at the imine position, which decreases the basicity of the imine by the delocalization of the nitro­gen lone pair *p*-orbital into the aromatic group, facilitating isolation (Guest *et al.*, 2020 ▸; Kozma *et al.*, 2015 ▸; Bruns *et al.*, 2010 ▸). Some of these (Guest *et al.*, 2020 ▸; Kozma *et al.*, 2015 ▸; Belding & Dudding, 2014 ▸) are bis­(cylopropenimine) variants of the famous ‘proton sponge’, 1,8-bis­(di­methyl­amino)­naphthalene and related classes of bifunctional Lewis superbases (Alder *et al.*, 1968 ▸). The only other example, to our knowledge, is an *N*-amino­substituted example, which also decreases the basicity of the nitro­gen lone pair by induction, a minor resonance structure delocalizing the double bond into the N—N contact, and, in the crystal structure, a nearby hydrogen bond with a water proton (Bruns *et al.*, 2010 ▸). 
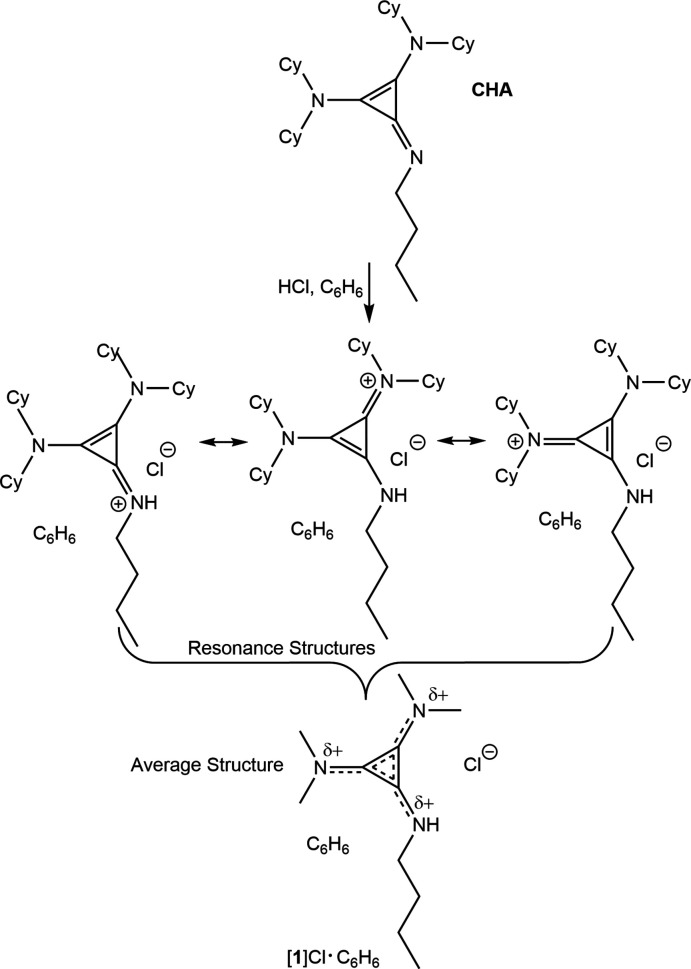


**Figure 1:**
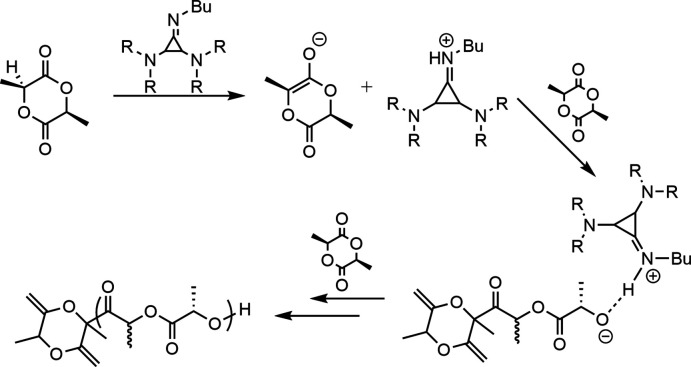
Catalytic ring-opening polymerization mediated by **1**.

*N*-Butyl-2,3-bis­(di­cyclo­hexyl­amino)­cyclo­propenimine (**1**) is a newer version of superbase with improved basicity, which has been explored as a catalyst for ring-opening polymerization. Cyclo­propenimines have a conjugate acid *pK_a_* of about 27, an improvement over that of the superbase 2-*tert*-butyl-1,1,3,3-tetra­methyl­guanidine (BTMG), which has a *pK_a_* of 23.56 (Bandar & Lambert, 2012 ▸). This allows **1** [Chem scheme1] to deprotonate a lactide and initiate polymerization in the synthesis of polylactic acid, as shown in Fig. 1 ▸ (Stukenbroeker *et al.*, 2015 ▸). Compound **1** can mediate the polymerization of lactic acid to 99% completion in 10 minutes or less. However, no X-ray crystal structure of the free base, nor an acid salt of this superbase has been reported. In this report we provide the first X-ray crystallographic structure of a benzene solvate of the hydro­chloride salt [**1**H]Cl·C_6_H_6_.

## 2. Structural commentary

**Figure 2:**
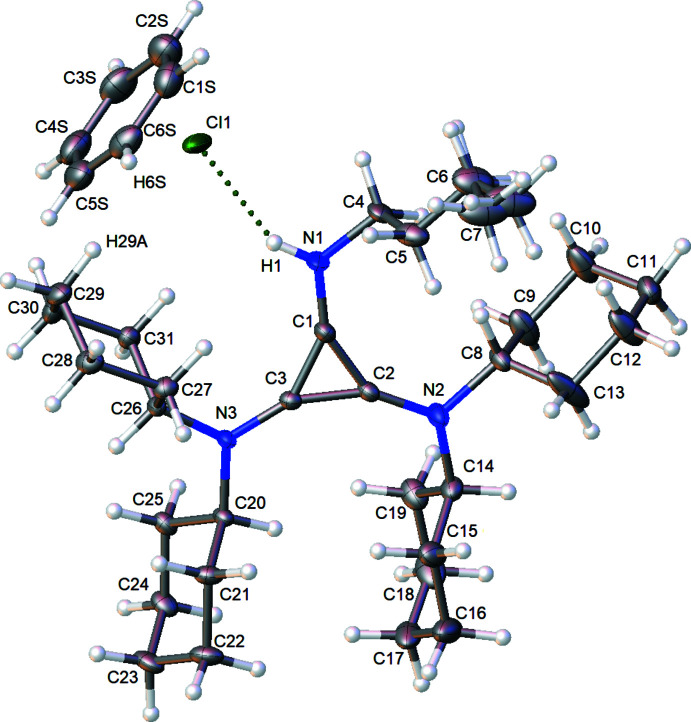
Displacement ellipsoid plot of the asymmetric unit of [**1**H]Cl·C_6_H_6_ with ellipsoids set at the 50% probability level. Hydrogen atoms shown as small spheres.

[**1**H]Cl crystallizes in the *P*2_1_/*n* space group on a general position as a closely associated ion pair, with the protonation site at the *n*-butyl imine as expected, and one formula unit in the asymmetric unit, as well as one benzene molecule, also on a general position (Fig. 2 ▸). The organic salt and the benzene molecule are generally well ordered, except for the δ methyl carbon of the *n*-butyl group, which shows a mild wagging disorder. This disorder was treated with a two-site disorder model.

Free-base **1** would be expected to have localized double bonds at the *n*-butyl­imine C=N position, and at the opposing cyclo­propene position (see scheme[Chem scheme1]). In the isolated free base of 1-mesityl-2,3-bis­(diiso­propyl­amino)­cyclo­propenimine (Bruns *et al.*, 2010 ▸), the unprotonated C=N imine bond is 1.2951 (14) Å in length, while the C—N bonds to the tertiary amines are longer, at an average of 1.3494 (10) Å. The localized cyclo­propene double bond is shorter, at 1.3712 (14) Å, than the single bonded C—C cyclo­propene contacts at an average of 1.4155 (10) Å. Protonation of the *n*-butyl­imine position during crystal growth results in all nitro­gen atoms being three-coordinate, leading to delocalization of the imine double-bond character across all three C—N contacts. Correspondingly, the cyclo­propene double bond is delocalized around the ring, giving a three-membered aromatic system. In [**1**H]Cl, the central C_3_N_3_ triangle is thus highly planar, with the six atoms exhibiting an r.m.s. deviation of only 0.0052 Å from the best-fit plane of the six atoms. The three C—N bonds are approximately equal in length, with the two tertiary cyclo­hexyl­amine positions having C—N lengths of 1.3279 (13) Å on average. The C—N bond to the protonated butyl nitro­gen is only slightly shorter at 1.319 (2) Å. The three cyclo­propene C—C bonds exhibit lengths consistent with aromaticity; the unique C—C bond opposite the *n*-butyl group is 1.388 (2) Å, while the other two C—C bonds are similar or slightly shorter at 1.377 (2) and 1.383 (2) Å. Though these latter two bonds are equivalent under mol­ecular point symmetry, their differences are attributed to the asymmetric crystal packing environment of the *P*2_1_/*n* space group. The comparable nature of the bond metrics of the three C—N bonds and the three cyclo­propenyl C—C bonds is consistent with aromatization, and an analogous aromatization of the C_3_N_3_ core of 1-mesityl-2,3-bis­(diiso­propyl­amino)­cyclo­propeniminium tetra­fluoro­borate was observed in the crystal structure of this salt (Bruns *et al.*, 2010 ▸). See Table 1 ▸ for C_3_N_3_ bond metrics.

The comparison between free-base forms of cyclo­propenimine (Bruns *et al.*, 2010 ▸) and the protonated forms demonstrate aromatization upon protonation, and explain the behavior of **1** as a superbase. While alkyl­imines are typically weak bases (*pK_a_* of conjugate acid ranges from about 2–5 (Fraser *et al.*, 1983 ▸), the *pK_a_* of **1**H^+^ is a staggering 27 (Bandar & Lambert, 2012 ▸), more on the scale of a C—H bond. The drastic difference in basicity between typical alkyl­imines and **1** can be explained by the observed aromatization upon proton­ation. As a result, the ^1^H resonance of the N—H hydrogen in [**1**H]Cl is a sharp singlet at 7.4 ppm in deuterated chloro­form, suggesting little to no exchange like that typically observed for broad N—H resonances. The stabilization of a mol­ecule by aromatization is qu­anti­fied by the Dewar Resonance Energy (DRE), which ranges from about 6–25 kJ mol^−1^ per π electron (Slayden & Liebman, 2001 ▸). Thus in the case of **1**, aromatic stabilization between 12 and 50 kJ mol^−1^ upon protonation explains the large reported basicity.

## 3. Supra­molecular features

**Figure 3:**
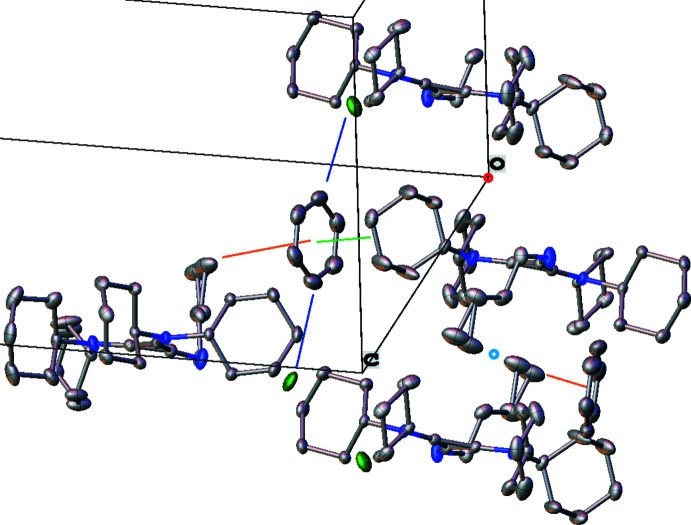
Partially packed thermal ellipsoid plot of [**1**H]Cl·C_6_H_6_ showing neighboring inter­molecular/inter­ionic nearest neighbor inter­actions.

**Figure 4:**
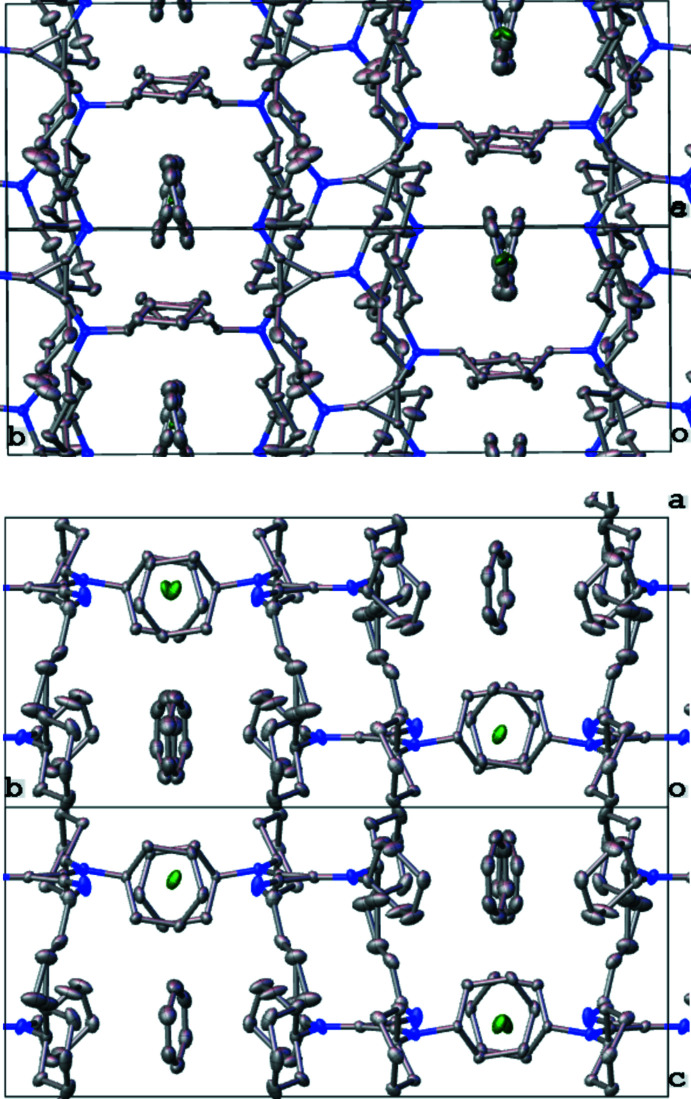
Top: Packed unit cell viewed along the 101 plane. Bottom: Packed unit cell viewed along the [101] direction.

Inter­ionic/mol­ecular inter­actions were examined using packing diagrams, and by the determination of partial atomic charge from Hirshfeld analysis. In the following discussion Hirshfeld charges are presented in parenthesis. The proton of the butyl­imine group (+0.121) inter­acts strongly with the chloride ion (−0.666) at a short H⋯Cl distance of 2.26 (2) Å. The chloride is positioned in a pocket surrounded by hydrogen atoms. In addition to the strong inter­action with the acidic N—H proton, the chloride resides 2.8152 (7) Å from a benzene proton, H6*AA* (+0.046), and 2.7169 (6) Å from an intra­molecular axial cyclo­hexyl proton, H29*A* (+0.050). The crystal packing demonstrates that the C_3_N_3_ planes of all mol­ecules pack parallel to each other (as required by the space-group symmetry), with a normal slightly oblique to the (101) plane (see Fig. 3 ▸). The mol­ecular planes stack in a staggered fashion *via* inter­vening inversion centers at the origin (Fig. 3 ▸, red) and at the center of the *a* edge (Fig. 3 ▸, teal). One face of the benzene solvent molecule inter­acts distally with the cyclo­hexyl group of one 1H^+^ ion [closest atomic C⋯C distance: 3.829 (3) Å, Fig. 3 ▸, green line], while the other face inter­acts distally with the disordered methyl group of the *n*-butyl chain [closest atomic C⋯C distance: 4.29 (3) Å, Fig. 3 ▸, orange line]. The benzene inter­acts weakly with two chloride ions approximately along its equatorial plane (Fig. 3 ▸, blue lines), one *via* H6*S* (+0.069) with H⋯Cl = 2.8152 (7) Å, also shown in Fig. 2 ▸, and the other *via* H3*S* (+0.062) with H⋯Cl = 2.8365 (7) Å. These benzene–chlorine inter­actions form a channel along the (101) plane, each channel situated 1/4 of the way along the *b* axis (Fig. 4 ▸, top). Viewed from 90° along the [101] direction, the benzene solvent molecules sit along a second channel, with the chloride ions sitting at the inter­sections of both channels, providing ionic bonds to the surrounding 1H^+^ cations (Fig. 4 ▸, bottom). In this latter view, it is apparent that along the [101] direction, the chloride ions are positioned between the axial protons H26 (+0.058) and H29*B* (+0.059) of the flanking cyclo­hexyl groups. In summary, the **1**H^+^ cations inter­act with each other and through the benzene solvent molecule *via* their alkyl groups, and the chloride counter-ion is situated in a pocket of cyclo­hexyl and benzene C—H contacts, with the proximal N—H inter­action on one side.

## 4. Database survey

In addition to the penta­substituted examples discussed above, a survey of the Cambridge Structural Database (CSD, Version 5.34, November 2021; Groom *et al.*, 2016 ▸) for cyclo­propenimines reveals a number of other relevant structures. The parent (unsubstituted) di­amino­propeniminium cation has been structurally characterized with chloride and iodide counter-cations (UJAVEI and UJAVIM; Mishiro *et al.*, 2016 ▸). Aprotic hexa­substituted examples are reported, and represent planar polyatomic cations (AHUVEH, Holthoff *et al.*, 2020 ▸; DOSRUB, Abdelbassit *et al.*, 2019 ▸; FURCIH, Clark *et al.*, 1995 ▸; GAXYEJ, Radhakrishnan *et al.*, 1987 **b* ▸;* GERXUX02, Butchard *et al.*, 2012 ▸; GUNDUR, Curnow & Senthooran, 2020 ▸; IFAGUU, Curnow *et al.*, 2018 ▸; LAYYOC01, Jin *et al.*, 2018 ▸; NUYBOB, Guest *et al.*, 2020 ▸; SERVIW, Kniep *et al.*, 2013 ▸; TUSDOD, Radhakrishnan *et al.*, 1987*a* ▸; UGITIQ, Barthes *et al.*, 2020 ▸, XIKYAT01, ZABFUG, Wallace *et al.*, 2015 ▸, XOSTIL, XOSTOR, XOSTUX, XOSVAF, XOSVEJ, Abdelbassit & Curnow, 2019 ▸, YUVRAK, YUWJOR, Jungbauer *et al.*, 2015 ▸). Another class of variants includes cyclo­propenemines tethered to ferrocene nuclei (TURNUQ, Bruns *et al.*, 2010 ▸; BEBPIK, BEBRAE, BEBREI, BEBRIM, BEBROS, Jess *et al.*, 2017 ▸). There are a few structural studies of Lewis complexes with metal ions (BEBRIM, Jess *et al.*, 2017 ▸; UGITOW, UGITUC, Barthes *et al.*, 2020 ▸; YOQPOM, Chen *et al.*, 2019 ▸; TURNOK, Bruns *et al.*, 2010 ▸) or other boron-based Lewis acids (NUYBOB, Guest *et al.*, 2020 ▸; TURPOM, Bruns *et al.*, 2010 ▸). One structural report of a tris­ubstituted cyclo­propenimine is noted (XEXGEP; Xu *et al.*, 2018 ▸), as well as several types of oligomeric versions (OGOLUT, OGORAF, OGOWOY, OGOWUE, OGOXAL, OGOXEP, OGOXIT, OGOXOZ, OGOXUF, OGOYAM, Kozma *et al.*, 2015 ▸; SUSWAG, SUSWOU, Nacsa & Lambert, 2015 ▸).

## 5. Synthesis and crystallization

Initially, crystals of [**1**H]Cl·C_6_H_6_ were obtained from the commercial sample of **1** *via* a double-vial apparatus by dissolution of *N*-butyl-2,3-bis­(di­cyclo­hexyl­amino)­cyclo­prop­enimine (**1**) in benzene in an inner vial, and charging the outer vial with hexa­nes. After diffusion for a few days at room temperature, powdery solid and a few colorless crystals of [**1**H]Cl·C_6_H_6_ were observed inside. The yield of crystalline [**1**H]Cl·C_6_H_6_ was significantly improved by the addition of HCl. To a glass shell vial containing 7.2 mg of *N*-butyl-2,3-bis­(di­cyclo­hexyl­amino)­cyclo­propenimine, 2 mL of benzene were added. A drop of dilute HCl (0.730 *M*) was added. This was diffused with 3 mL of hexa­nes in the outer vial for 2–3 days. Crystallization works best when the drop is not in contact with the walls of the vial where the crystals grow. Crystals were isolated by deca­nting the liquid from the inner vial using a disposable pipette, and taking care to remove the visible aqueous HCl droplet with the first pipette draw. After removing the mother liquor, the crystals were rinsed with hexa­nes. Yield 6.3 mg (70%). Yields in this small-scale preparation ranged from 22% to 70% across multiple attempts. ^1^H NMR (ppm) 400 MHz, CDCl_3_): δ(ppm): 0.97 (*t*, 3H, Me), 1.62–1.82 (*m*, 14H, Cy and Bu), 1.62–1.76 (*m*, 14H, Cy and Bu), 1.80 (*d*, 8H, Cy-β-H), 1.96 (*d*, 8H, Cy-β-H), 3.34 (*tt*, 4H, Cy-α-H), 3.56 (t, 2H, Bu-α-H), 7.4 (s, 1H, NH). ^13^C NMR (ppm) (400 MHz, CDCl_3_): δ(ppm): 13.97, 19.94, 24.58, 25.84, 32.34, 33.79, 46.15, 59.55, 114.01, 128.35. FTIR (cm^−1^): 2926 (*m*), 2851 (*m*), 1503 (*s*), 1445 (*m*), 1383 (*w*), 1374 (*w*), 1345 (*w*), 1324 (*w*), 1253 (*w*), 1188 (*w*), 1180 (*w*), 1102 (*w*), 1092 (*w*), 1004 (*w*), 895 (*w*), 696 (*m*). Analysis calculated for C_31_H_53_N_3_·0.5 C_6_H_6_ (%): C, 76.31; H, 10.38; N, 7.22. Found: C, 75.873; H, 10.83; N, 7.24. M.p. 353–356 K (decomposes).

## 6. Refinement

Crystal data, data collection and structure refinement details are summarized in Table 2 ▸. A disordered methyl group was treated with a two-site disorder model, with atom positions freely refined, and relative occupancies refined using Free Variable 2 with a final ratio of 0.71 (3): 0.29 (3). RIGU/SIMU restraints were applied to the wagging methyl group. C—H hydrogen atoms were treated using a standard riding model. The imine proton was located as a peak in the Fourier difference map and was freely refined.

Hirshfeld charge was determined at the 3-21G/B3LYP level of theory by iterative computation of electronic structure of [**1**H]Cl·C_6_H_6_ using ORCA (Neese, 2018 ▸) followed by rerefinement of the structure using non-spherical form factors computed using *NoSpherA2* (Kleemiss *et al.*, 2021 ▸), and repeating the process until the structure converged. Hirshfeld charges resulting from this approach are given in Table 3 ▸.

## Supplementary Material

Crystal structure: contains datablock(s) I. DOI: 10.1107/S2056989022008076/jq2013sup1.cif

Structure factors: contains datablock(s) I. DOI: 10.1107/S2056989022008076/jq2013Isup2.hkl

Supporting information file. DOI: 10.1107/S2056989022008076/jq2013Isup3.cdx

CCDC reference: 2200141

Additional supporting information:  crystallographic information; 3D view; checkCIF report

## Figures and Tables

**Table 1:** Comparative bond lengths (Å) for 1-mesityl-2,3-bis­(diiso­propyl­amino)­cyclo­propenimine, 1-mesityl-2,3-bis­(diiso­propyl­amino)­cyclo­propeniminium (Bruns *et al.*, 2010 ▸), and *N*-*n*-butyl-2,3-bis­(di­cyclo­hex­yl)cyclo­propeniminium Divided entries refer to separate, related pairs of atoms and their associated metrics, *e.g.*, 1.3450 (14)/1.3539 (14) denotes two distances for the two C—N(amine) contacts.

	Mes(C_3_N_3_)^*i*^Pr_4_	[Mes(C_3_N_3_H)^*i*^Pr_4_]BF_4_	[Bu(C_3_N_3_H)Cy_4_]Cl([**1** *H*]Cl)
C—N(imine)	1.2951 (14)	1.3342 (16)	1.319 (2)
C—N(amine)	1.3450 (14)/1.3539 (14)	1.3205 (15)/1.3286 (16)	1.3248 (17)/1.331 (2)
C—C(*para*)	1.3712 (14)	1.3984 (17)	1.388 (2)
C—C(*meta*)	1.4202 (14)/1.4108 (14)	1.3792 (16)/1.3827 (16)	1.377 (2)/1.3831 (19)

**Table 2:** Experimental details

Crystal data	
Chemical formula	C_31_H_54_N_3_^+^·Cl^−^·C_6_H_6_
*M*_r_	582.33
Crystal system, space group	Monoclinic, *P*2_1_/*n*
Temperature (K)	100
*a*, *b*, *c* (Å)	12.253 (3), 22.699 (7), 12.884 (3)
β (°)	104.164 (7)
*V* (Å^3^)	3474.6 (16)
*Z*	4
Radiation type	Mo *K*α
μ (mm^−1^)	0.14
Crystal size (mm)	0.55 × 0.53 × 0.16

Data collection	
Diffractometer	Bruker D8 Quest Photon 100
Absorption correction	Multi-scan (*SADABS*; Krause *et al.*, 2015 ▸)
*T*_min_, *T*_max_	0.662, 0.746
No. of measured, independent and observed [*I* > 2σ(*I*)] reflections	48756, 8072, 6516
*R*_int_	0.044
(sin θ/λ)_max_ (Å^−1^)	0.658

Refinement	
*R*[*F* ^2^ > 2σ(*F* ^2^)], *wR*(*F* ^2^), *S*	0.055, 0.136, 1.02
No. of reflections	8072
No. of parameters	385
No. of restraints	6
H-atom treatment	H atoms treated by a mixture of independent and constrained refinement
Δρ_max_, Δρ_min_ (e Å^−3^)	0.55, −0.40

Computer programs: *COSMO*, *XPREP*, and *SAINT* (Bruker, 2008 ▸), *SHELXT* (Sheldrick, 2015*a* ▸), *SHELXL* (Sheldrick, 2015*b* ▸), and *OLEX2* (Dolomanov *et al.*, 2009 ▸).

**Table 3:** Hirshfeld charges of atoms in [**1**H]Cl·C_6_H_6_.

Cl1	−0.666	N1	−0.048	N2	−0.027
N3	−0.018	C1	0.026	C2	0.014
C3	0.024	C4	−0.036	C5	−0.102
C6	−0.095	C7	−0.133	C8	−0.011
C9	−0.103	C10	−0.093	C11	−0.097
C12	−0.088	C13	−0.093	C14	−0.008
C15	−0.097	C16	−0.094	C17	−0.098
C18	−0.098	C19	−0.101	C20	−0.005
C21	−0.094	C22	−0.093	C23	−0.093
C24	−0.091	C25	−0.093	C26	−0.002
C27	−0.096	C28	−0.094	C29	−0.099
C30	−0.093	C31	−0.097	C1*S*	−0.058
C2*S*	−0.071	C3*S*	−0.078	C4*S*	−0.084
C5*S*	−0.082	C6*S*	−0.064	H1*S*	0.071
H6*S*	0.069	H5*S*	0.065	H4*S*	0.062
H3*S*	0.062	H2*S*	0.056	H4*A*	0.051
H4*B*	0.075	H5*A*	0.061	H5b	0.042
H6*AA*	0.046	H6*AB*	0.055	H7*A*	0.050
H7*B*	0.039	H7*C*	0.051	H8	0.072
H9*A*	0.050	H9*B*	0.060	H10*A*	0.061
H10*B*	0.061	H11*A*	0.061	H11*B*	0.049
H12*A*	0.057	H12*B*	0.053	H13*A*	0.059
H13*B*	0.049	H14	0.066	H15*A*	0.057
H15*B*	0.062	H16*A*	0.051	H16*B*	0.052
H17*A*	0.055	H17*B*	0.051	H18*A*	0.056
H18*B*	0.062	H19*A*	0.055	H19*B*	0.065
H20	0.060	H21*A*	0.055	H21*B*	0.055
H22*A*	0.050	H22*B*	0.056	H23*A*	0.056
H23*B*	0.052	H24*A*	0.051	H24*B*	0.056
H25*A*	0.057	H25*B*	0.057	H26	0.058
H27*A*	0.049	H27*B*	0.062	H28*A*	0.044
H28*B*	0.057	H29*A*	0.050	H29*B*	0.059
H30*A*	0.061	H30*B*	0.039	H31*A*	0.047
H31*B*	0.064	H1	0.121		

## supplementary crystallographic information

### Crystal data

**Table:**

C_31_H_54_N_3_^+^·Cl^−^·C_6_H_6_	*D*_x_ = 1.113 Mg m^−^^3^
*M**_r_* = 582.33	Melting point: 356 K
Monoclinic, *P*2_1_/*n*	Mo *K*α radiation, λ = 0.71076 Å
*a* = 12.253 (3) Å	Cell parameters from 9959 reflections
*b* = 22.699 (7) Å	θ = 2.6–29.6°
*c* = 12.884 (3) Å	µ = 0.14 mm^−^^1^
β = 104.164 (7)°	*T* = 100 K
*V* = 3474.6 (16) Å^3^	Chunk, colourless
*Z* = 4	0.55 × 0.53 × 0.16 mm
*F*(000) = 1280	

### Data collection

**Table:**

Bruker D8 Quest Photon 100 diffractometer	8072 independent reflections
Radiation source: sealed tube	6516 reflections with *I* > 2σ(*I*)
Detector resolution: 10.417 pixels mm^-1^	*R*_int_ = 0.044
φ and ω scans	θ_max_ = 27.9°, θ_min_ = 2.6°
Absorption correction: multi-scan (SADABS; Krause *et al.*, 2015)	*h* = −16→14
*T*_min_ = 0.662, *T*_max_ = 0.746	*k* = −29→29
48756 measured reflections	*l* = −16→16

### Refinement

**Table:**

Refinement on *F*^2^	Secondary atom site location: difference Fourier map
Least-squares matrix: full	Hydrogen site location: mixed
*R*[*F*^2^ > 2σ(*F*^2^)] = 0.055	H atoms treated by a mixture of independent and constrained refinement
*wR*(*F*^2^) = 0.136	*w* = 1/[σ^2^(*F*_o_^2^) + (0.0605*P*)^2^ + 2.3777*P*] where *P* = (*F*_o_^2^ + 2*F*_c_^2^)/3
*S* = 1.02	(Δ/σ)_max_ = 0.001
8072 reflections	Δρ_max_ = 0.55 e Å^−^^3^
385 parameters	Δρ_min_ = −0.40 e Å^−^^3^
6 restraints	Extinction correction: SHELXL2018/3 (Sheldrick 2015b), Fc^*^=kFc[1+0.001xFc^2^λ^3^/sin(2θ)]^-1/4^
Primary atom site location: dual	Extinction coefficient: 0.0081 (7)

### Special details

**Table:**

Experimental. Single-crystal X-ray crystallographic data were obtained on a Bruker D8 Quest PHOTON 100 diffractometer with an Oxford Cryostream 700 low-temperature device. The radiation was from a sealed-tube molybdenum *K*α source with a TRIUMPH monochromator. Crystals were typically multiple, and a single piece was cut away with a razor blade, mounted on a MiTeGen loop with paratone-N oil, and collected at 100K in ω/φ scansets. Integration was performed using SAINT, and data were reduced and absorption-corrected using SADABS (Bruker, 2008). Space group determination was performed using XPREP (Sheldrick, 2008), and the structure was solved using intrinsic phasing using SHELXT (Sheldrick,The structural model of [1H]Cl·C_6_H_6_ was refined using the least-squares approach with the ShelX package (Sheldrick, 2015a) with Olex2 as a GUI (Dolomanov *et al.*, 2009). 2015b).
Geometry. All esds (except the esd in the dihedral angle between two l.s. planes) are estimated using the full covariance matrix. The cell esds are taken into account individually in the estimation of esds in distances, angles and torsion angles; correlations between esds in cell parameters are only used when they are defined by crystal symmetry. An approximate (isotropic) treatment of cell esds is used for estimating esds involving l.s. planes.

### Fractional atomic coordinates and isotropic or equivalent isotropic displacement parameters (Å^2^)

**Table:**

	*x*	*y*	*z*	*U*_iso_*/*U*_eq_	Occ. (<1)
Cl1	0.44296 (4)	0.74552 (2)	0.69373 (3)	0.03152 (13)	
N1	0.36809 (13)	0.61997 (7)	0.64021 (11)	0.0287 (3)	
N2	0.28059 (11)	0.47671 (6)	0.52228 (11)	0.0214 (3)	
N3	0.16710 (10)	0.61804 (5)	0.38294 (10)	0.0153 (3)	
C1	0.30625 (12)	0.58844 (7)	0.56140 (12)	0.0187 (3)	
C2	0.27226 (12)	0.53505 (7)	0.51360 (11)	0.0161 (3)	
C3	0.23000 (12)	0.58703 (6)	0.46257 (11)	0.0152 (3)	
C4	0.42658 (14)	0.59655 (8)	0.74386 (13)	0.0258 (4)	
H4A	0.482194	0.625847	0.781488	0.031*	
H4B	0.468020	0.560497	0.733256	0.031*	
C5	0.34533 (15)	0.58187 (9)	0.81298 (14)	0.0297 (4)	
H5A	0.299691	0.617193	0.818508	0.036*	
H5B	0.293374	0.550454	0.777551	0.036*	
C6	0.40470 (18)	0.56176 (11)	0.92421 (15)	0.0417 (5)	
H6AA	0.453690	0.528001	0.917569	0.050*	0.71 (3)
H6AB	0.454139	0.594103	0.959793	0.050*	0.71 (3)
H6BC	0.463745	0.589993	0.959547	0.050*	0.29 (3)
H6BD	0.438414	0.522186	0.923334	0.050*	0.29 (3)
C7	0.3303 (10)	0.5437 (7)	0.9956 (7)	0.053 (2)	0.71 (3)
H7A	0.376946	0.531504	1.065397	0.079*	0.71 (3)
H7B	0.282871	0.577033	1.005181	0.079*	0.71 (3)
H7C	0.282420	0.510733	0.962831	0.079*	0.71 (3)
C7A	0.302 (2)	0.5607 (13)	0.980 (2)	0.052 (3)	0.29 (3)
H7AA	0.329164	0.548113	1.055049	0.079*	0.29 (3)
H7AB	0.269538	0.600292	0.978288	0.079*	0.29 (3)
H7AC	0.244456	0.533150	0.942430	0.079*	0.29 (3)
C8	0.36221 (13)	0.45256 (7)	0.61774 (12)	0.0217 (3)	
H8	0.381382	0.485489	0.670631	0.026*	
C9	0.47084 (15)	0.43290 (10)	0.59447 (16)	0.0353 (4)	
H9A	0.502862	0.465328	0.559880	0.042*	
H9B	0.456679	0.398984	0.544653	0.042*	
C10	0.55473 (15)	0.41505 (11)	0.69901 (17)	0.0402 (5)	
H10A	0.624144	0.399694	0.682382	0.048*	
H10B	0.575268	0.450269	0.744965	0.048*	
C11	0.50711 (16)	0.36903 (8)	0.75884 (15)	0.0304 (4)	
H11A	0.560159	0.362242	0.829220	0.036*	
H11B	0.499093	0.331562	0.718347	0.036*	
C12	0.39465 (16)	0.38660 (12)	0.77577 (17)	0.0443 (6)	
H12A	0.404691	0.420050	0.826542	0.053*	
H12B	0.362632	0.353219	0.807993	0.053*	
C13	0.31273 (15)	0.40433 (13)	0.67130 (17)	0.0502 (7)	
H13A	0.295939	0.369777	0.623135	0.060*	
H13B	0.241307	0.418020	0.686061	0.060*	
C14	0.22900 (14)	0.43733 (7)	0.43423 (12)	0.0228 (3)	
H14	0.251060	0.396403	0.459615	0.027*	
C15	0.10137 (15)	0.43961 (8)	0.40904 (14)	0.0294 (4)	
H15A	0.075663	0.480402	0.389687	0.035*	
H15B	0.075736	0.428155	0.473345	0.035*	
C16	0.04928 (19)	0.39800 (10)	0.31628 (16)	0.0421 (5)	
H16A	0.065028	0.356690	0.339859	0.051*	
H16B	−0.033395	0.403439	0.296143	0.051*	
C17	0.09633 (19)	0.40960 (8)	0.21891 (15)	0.0388 (5)	
H17A	0.064423	0.380550	0.162311	0.047*	
H17B	0.073038	0.449374	0.190386	0.047*	
C18	0.2230 (2)	0.40551 (9)	0.24633 (15)	0.0404 (5)	
H18A	0.250416	0.415061	0.182158	0.048*	
H18B	0.246121	0.364657	0.268072	0.048*	
C19	0.27602 (15)	0.44745 (8)	0.33640 (13)	0.0281 (4)	
H19A	0.261060	0.488590	0.311459	0.034*	
H19B	0.358567	0.441498	0.356404	0.034*	
C20	0.10054 (12)	0.59000 (6)	0.28437 (11)	0.0154 (3)	
H20	0.121932	0.547404	0.287367	0.018*	
C21	−0.02557 (12)	0.59316 (7)	0.27777 (12)	0.0208 (3)	
H21A	−0.048940	0.634910	0.277906	0.025*	
H21B	−0.041475	0.573704	0.341256	0.025*	
C22	−0.09312 (14)	0.56299 (8)	0.17598 (14)	0.0270 (4)	
H22A	−0.076043	0.520300	0.179794	0.032*	
H22B	−0.174555	0.567825	0.170890	0.032*	
C23	−0.06516 (14)	0.58893 (8)	0.07659 (13)	0.0273 (4)	
H23A	−0.106868	0.567204	0.012491	0.033*	
H23B	−0.089479	0.630630	0.068675	0.033*	
C24	0.06045 (14)	0.58532 (8)	0.08384 (12)	0.0232 (3)	
H24A	0.076785	0.604027	0.019878	0.028*	
H24B	0.083535	0.543480	0.085098	0.028*	
C25	0.12775 (12)	0.61616 (7)	0.18448 (12)	0.0188 (3)	
H25A	0.109634	0.658734	0.180255	0.023*	
H25B	0.209217	0.611798	0.189237	0.023*	
C26	0.15551 (12)	0.68225 (6)	0.39646 (12)	0.0155 (3)	
H26	0.099398	0.696644	0.331400	0.019*	
C27	0.10861 (13)	0.69733 (7)	0.49275 (12)	0.0198 (3)	
H27A	0.037198	0.675821	0.487607	0.024*	
H27B	0.162771	0.684987	0.559541	0.024*	
C28	0.08784 (14)	0.76347 (7)	0.49579 (14)	0.0235 (3)	
H28A	0.028444	0.774989	0.432041	0.028*	
H28B	0.060970	0.773161	0.560235	0.028*	
C29	0.19481 (14)	0.79815 (7)	0.49779 (14)	0.0258 (3)	
H29A	0.251074	0.790334	0.565857	0.031*	
H29B	0.177627	0.840825	0.494357	0.031*	
C30	0.24396 (14)	0.78122 (7)	0.40405 (14)	0.0236 (3)	
H30A	0.315816	0.802469	0.410204	0.028*	
H30B	0.191359	0.793486	0.336272	0.028*	
C31	0.26484 (12)	0.71506 (7)	0.40078 (12)	0.0190 (3)	
H31A	0.292722	0.705234	0.336957	0.023*	
H31B	0.322722	0.703020	0.465293	0.023*	
C1S	0.68908 (19)	0.74647 (9)	0.54411 (18)	0.0396 (5)	
H1S	0.696225	0.738475	0.617844	0.048*	
C2S	0.78338 (18)	0.74472 (10)	0.5024 (2)	0.0445 (5)	
H2S	0.855070	0.735644	0.547561	0.053*	
C3S	0.77266 (19)	0.75618 (10)	0.3953 (2)	0.0430 (5)	
H3S	0.836697	0.754721	0.366155	0.052*	
C4S	0.6681 (2)	0.76980 (9)	0.33078 (18)	0.0414 (5)	
H4S	0.660378	0.777832	0.256966	0.050*	
C5S	0.57486 (18)	0.77183 (9)	0.37275 (18)	0.0390 (5)	
H5S	0.503360	0.781612	0.327955	0.047*	
C6S	0.58513 (17)	0.75981 (8)	0.47859 (18)	0.0367 (4)	
H6S	0.520582	0.760638	0.507068	0.044*	
H1	0.3710 (18)	0.6567 (11)	0.6337 (18)	0.035 (6)*	

### Atomic displacement parameters (Å^2^)

**Table:**

	*U*^11^	*U*^22^	*U*^33^	*U*^12^	*U*^13^	*U*^23^
Cl1	0.0369 (2)	0.0277 (2)	0.0232 (2)	−0.01656 (17)	−0.00559 (16)	−0.00049 (16)
N1	0.0371 (8)	0.0193 (7)	0.0193 (7)	−0.0048 (6)	−0.0128 (6)	0.0002 (6)
N2	0.0270 (7)	0.0164 (6)	0.0174 (6)	0.0060 (5)	−0.0013 (5)	−0.0020 (5)
N3	0.0169 (6)	0.0138 (6)	0.0123 (6)	0.0014 (5)	−0.0021 (5)	−0.0010 (5)
C1	0.0179 (7)	0.0185 (7)	0.0166 (7)	0.0012 (6)	−0.0017 (6)	0.0013 (6)
C2	0.0153 (6)	0.0185 (7)	0.0129 (6)	0.0028 (5)	0.0003 (5)	−0.0009 (5)
C3	0.0142 (6)	0.0168 (7)	0.0138 (7)	−0.0005 (5)	0.0017 (5)	−0.0023 (5)
C4	0.0286 (8)	0.0260 (8)	0.0159 (7)	−0.0020 (7)	−0.0081 (6)	0.0003 (6)
C5	0.0251 (8)	0.0343 (10)	0.0262 (9)	0.0035 (7)	−0.0007 (7)	−0.0116 (7)
C6	0.0414 (11)	0.0616 (14)	0.0217 (9)	−0.0093 (10)	0.0068 (8)	−0.0050 (9)
C7	0.044 (4)	0.091 (6)	0.026 (3)	−0.019 (3)	0.012 (2)	−0.012 (3)
C7A	0.047 (7)	0.085 (8)	0.029 (5)	−0.024 (5)	0.016 (5)	−0.017 (5)
C8	0.0245 (8)	0.0200 (7)	0.0167 (7)	0.0071 (6)	−0.0023 (6)	−0.0010 (6)
C9	0.0238 (8)	0.0507 (12)	0.0337 (10)	0.0075 (8)	0.0112 (7)	0.0191 (9)
C10	0.0218 (8)	0.0586 (13)	0.0395 (11)	0.0113 (8)	0.0063 (8)	0.0230 (10)
C11	0.0353 (9)	0.0249 (9)	0.0254 (9)	0.0039 (7)	−0.0033 (7)	0.0046 (7)
C12	0.0296 (9)	0.0728 (16)	0.0298 (10)	0.0027 (10)	0.0060 (8)	0.0271 (10)
C13	0.0189 (8)	0.102 (2)	0.0284 (10)	−0.0030 (10)	0.0027 (7)	0.0339 (12)
C14	0.0334 (8)	0.0158 (7)	0.0166 (7)	−0.0001 (6)	0.0011 (6)	−0.0021 (6)
C15	0.0323 (9)	0.0330 (9)	0.0198 (8)	−0.0072 (7)	0.0003 (7)	0.0049 (7)
C16	0.0522 (12)	0.0348 (10)	0.0292 (10)	−0.0185 (9)	−0.0096 (9)	0.0069 (8)
C17	0.0635 (13)	0.0220 (9)	0.0205 (8)	−0.0053 (9)	−0.0096 (8)	−0.0035 (7)
C18	0.0667 (14)	0.0304 (10)	0.0201 (8)	0.0127 (9)	0.0030 (9)	−0.0075 (7)
C19	0.0349 (9)	0.0287 (9)	0.0205 (8)	0.0107 (7)	0.0063 (7)	−0.0022 (7)
C20	0.0157 (6)	0.0151 (7)	0.0127 (6)	0.0023 (5)	−0.0013 (5)	−0.0021 (5)
C21	0.0162 (7)	0.0268 (8)	0.0178 (7)	−0.0023 (6)	0.0007 (6)	−0.0062 (6)
C22	0.0175 (7)	0.0349 (9)	0.0251 (8)	−0.0024 (7)	−0.0015 (6)	−0.0099 (7)
C23	0.0243 (8)	0.0332 (9)	0.0182 (8)	0.0062 (7)	−0.0069 (6)	−0.0058 (7)
C24	0.0270 (8)	0.0277 (8)	0.0129 (7)	0.0058 (7)	0.0010 (6)	−0.0021 (6)
C25	0.0172 (7)	0.0231 (8)	0.0151 (7)	0.0043 (6)	0.0019 (6)	0.0003 (6)
C26	0.0170 (6)	0.0125 (7)	0.0152 (7)	0.0013 (5)	0.0005 (5)	−0.0006 (5)
C27	0.0213 (7)	0.0195 (7)	0.0185 (7)	−0.0010 (6)	0.0046 (6)	−0.0036 (6)
C28	0.0232 (8)	0.0216 (8)	0.0248 (8)	0.0012 (6)	0.0042 (6)	−0.0067 (6)
C29	0.0271 (8)	0.0170 (7)	0.0295 (9)	−0.0017 (6)	−0.0001 (7)	−0.0053 (6)
C30	0.0243 (8)	0.0164 (7)	0.0281 (8)	−0.0035 (6)	0.0024 (6)	0.0031 (6)
C31	0.0188 (7)	0.0183 (7)	0.0191 (7)	−0.0014 (6)	0.0032 (6)	0.0014 (6)
C1S	0.0548 (12)	0.0281 (10)	0.0371 (11)	−0.0012 (9)	0.0135 (9)	0.0017 (8)
C2S	0.0348 (10)	0.0405 (12)	0.0554 (14)	0.0020 (9)	0.0056 (10)	0.0035 (10)
C3S	0.0394 (11)	0.0378 (11)	0.0574 (14)	−0.0094 (9)	0.0229 (10)	−0.0042 (10)
C4S	0.0584 (13)	0.0277 (10)	0.0389 (11)	−0.0127 (9)	0.0137 (10)	−0.0011 (8)
C5S	0.0381 (10)	0.0241 (9)	0.0500 (12)	−0.0007 (8)	0.0010 (9)	−0.0026 (9)
C6S	0.0341 (10)	0.0261 (9)	0.0532 (13)	−0.0018 (7)	0.0171 (9)	−0.0077 (8)

### Geometric parameters (Å, º)

**Table:**

N1—C1	1.319 (2)	C16—C17	1.526 (3)
N1—C4	1.453 (2)	C17—H17A	0.9900
N1—H1	0.84 (2)	C17—H17B	0.9900
N2—C2	1.331 (2)	C17—C18	1.507 (3)
N2—C8	1.4872 (19)	C18—H18A	0.9900
N2—C14	1.461 (2)	C18—H18B	0.9900
N3—C3	1.3247 (19)	C18—C19	1.519 (3)
N3—C20	1.4752 (18)	C19—H19A	0.9900
N3—C26	1.4789 (19)	C19—H19B	0.9900
C1—C2	1.377 (2)	C20—H20	1.0000
C1—C3	1.383 (2)	C20—C21	1.528 (2)
C2—C3	1.388 (2)	C20—C25	1.527 (2)
C4—H4A	0.9900	C21—H21A	0.9900
C4—H4B	0.9900	C21—H21B	0.9900
C4—C5	1.526 (3)	C21—C22	1.531 (2)
C5—H5A	0.9900	C22—H22A	0.9900
C5—H5B	0.9900	C22—H22B	0.9900
C5—C6	1.510 (3)	C22—C23	1.523 (3)
C6—H6AA	0.9900	C23—H23A	0.9900
C6—H6AB	0.9900	C23—H23B	0.9900
C6—H6BC	0.9900	C23—C24	1.521 (2)
C6—H6BD	0.9900	C24—H24A	0.9900
C6—C7	1.502 (9)	C24—H24B	0.9900
C6—C7A	1.60 (2)	C24—C25	1.526 (2)
C7—H7A	0.9800	C25—H25A	0.9900
C7—H7B	0.9800	C25—H25B	0.9900
C7—H7C	0.9800	C26—H26	1.0000
C7A—H7AA	0.9800	C26—C27	1.528 (2)
C7A—H7AB	0.9800	C26—C31	1.522 (2)
C7A—H7AC	0.9800	C27—H27A	0.9900
C8—H8	1.0000	C27—H27B	0.9900
C8—C9	1.502 (2)	C27—C28	1.525 (2)
C8—C13	1.499 (3)	C28—H28A	0.9900
C9—H9A	0.9900	C28—H28B	0.9900
C9—H9B	0.9900	C28—C29	1.524 (2)
C9—C10	1.535 (3)	C29—H29A	0.9900
C10—H10A	0.9900	C29—H29B	0.9900
C10—H10B	0.9900	C29—C30	1.525 (2)
C10—C11	1.499 (3)	C30—H30A	0.9900
C11—H11A	0.9900	C30—H30B	0.9900
C11—H11B	0.9900	C30—C31	1.526 (2)
C11—C12	1.501 (3)	C31—H31A	0.9900
C12—H12A	0.9900	C31—H31B	0.9900
C12—H12B	0.9900	C1S—H1S	0.9500
C12—C13	1.523 (3)	C1S—C2S	1.389 (3)
C13—H13A	0.9900	C1S—C6S	1.379 (3)
C13—H13B	0.9900	C2S—H2S	0.9500
C14—H14	1.0000	C2S—C3S	1.378 (4)
C14—C15	1.518 (2)	C3S—H3S	0.9500
C14—C19	1.526 (2)	C3S—C4S	1.381 (3)
C15—H15A	0.9900	C4S—H4S	0.9500
C15—H15B	0.9900	C4S—C5S	1.379 (3)
C15—C16	1.535 (3)	C5S—H5S	0.9500
C16—H16A	0.9900	C5S—C6S	1.366 (3)
C16—H16B	0.9900	C6S—H6S	0.9500
			
C1—N1—C4	124.75 (15)	C16—C17—H17A	109.3
C1—N1—H1	119.5 (16)	C16—C17—H17B	109.3
C4—N1—H1	115.5 (16)	H17A—C17—H17B	107.9
C2—N2—C8	117.30 (13)	C18—C17—C16	111.77 (16)
C2—N2—C14	122.26 (13)	C18—C17—H17A	109.3
C14—N2—C8	119.49 (13)	C18—C17—H17B	109.3
C3—N3—C20	122.13 (12)	C17—C18—H18A	109.4
C3—N3—C26	119.14 (12)	C17—C18—H18B	109.4
C20—N3—C26	118.54 (11)	C17—C18—C19	111.36 (16)
N1—C1—C2	151.11 (15)	H18A—C18—H18B	108.0
N1—C1—C3	148.47 (15)	C19—C18—H18A	109.4
C2—C1—C3	60.37 (11)	C19—C18—H18B	109.4
N2—C2—C1	146.06 (14)	C14—C19—H19A	109.4
N2—C2—C3	153.88 (14)	C14—C19—H19B	109.4
C1—C2—C3	60.04 (11)	C18—C19—C14	111.05 (16)
N3—C3—C1	146.57 (14)	C18—C19—H19A	109.4
N3—C3—C2	153.84 (14)	C18—C19—H19B	109.4
C1—C3—C2	59.59 (11)	H19A—C19—H19B	108.0
N1—C4—H4A	109.3	N3—C20—H20	107.4
N1—C4—H4B	109.3	N3—C20—C21	111.47 (12)
N1—C4—C5	111.76 (15)	N3—C20—C25	111.67 (12)
H4A—C4—H4B	107.9	C21—C20—H20	107.4
C5—C4—H4A	109.3	C25—C20—H20	107.4
C5—C4—H4B	109.3	C25—C20—C21	111.18 (12)
C4—C5—H5A	109.0	C20—C21—H21A	109.5
C4—C5—H5B	109.0	C20—C21—H21B	109.5
H5A—C5—H5B	107.8	C20—C21—C22	110.73 (13)
C6—C5—C4	112.84 (15)	H21A—C21—H21B	108.1
C6—C5—H5A	109.0	C22—C21—H21A	109.5
C6—C5—H5B	109.0	C22—C21—H21B	109.5
C5—C6—H6AA	108.3	C21—C22—H22A	109.4
C5—C6—H6AB	108.3	C21—C22—H22B	109.4
C5—C6—H6BC	111.6	H22A—C22—H22B	108.0
C5—C6—H6BD	111.6	C23—C22—C21	111.21 (14)
C5—C6—C7A	100.6 (11)	C23—C22—H22A	109.4
H6AA—C6—H6AB	107.4	C23—C22—H22B	109.4
H6BC—C6—H6BD	109.4	C22—C23—H23A	109.4
C7—C6—C5	116.1 (5)	C22—C23—H23B	109.4
C7—C6—H6AA	108.3	H23A—C23—H23B	108.0
C7—C6—H6AB	108.3	C24—C23—C22	111.10 (13)
C7A—C6—H6BC	111.6	C24—C23—H23A	109.4
C7A—C6—H6BD	111.6	C24—C23—H23B	109.4
C6—C7—H7A	109.5	C23—C24—H24A	109.5
C6—C7—H7B	109.5	C23—C24—H24B	109.5
C6—C7—H7C	109.5	C23—C24—C25	110.84 (13)
H7A—C7—H7B	109.5	H24A—C24—H24B	108.1
H7A—C7—H7C	109.5	C25—C24—H24A	109.5
H7B—C7—H7C	109.5	C25—C24—H24B	109.5
C6—C7A—H7AA	109.5	C20—C25—H25A	109.5
C6—C7A—H7AB	109.5	C20—C25—H25B	109.5
C6—C7A—H7AC	109.5	C24—C25—C20	110.73 (13)
H7AA—C7A—H7AB	109.5	C24—C25—H25A	109.5
H7AA—C7A—H7AC	109.5	C24—C25—H25B	109.5
H7AB—C7A—H7AC	109.5	H25A—C25—H25B	108.1
N2—C8—H8	106.6	N3—C26—H26	106.8
N2—C8—C9	113.20 (14)	N3—C26—C27	112.48 (12)
N2—C8—C13	112.58 (14)	N3—C26—C31	112.18 (12)
C9—C8—H8	106.6	C27—C26—H26	106.8
C13—C8—H8	106.6	C31—C26—H26	106.8
C13—C8—C9	110.67 (16)	C31—C26—C27	111.30 (12)
C8—C9—H9A	109.7	C26—C27—H27A	109.7
C8—C9—H9B	109.7	C26—C27—H27B	109.7
C8—C9—C10	109.82 (15)	H27A—C27—H27B	108.2
H9A—C9—H9B	108.2	C28—C27—C26	109.86 (13)
C10—C9—H9A	109.7	C28—C27—H27A	109.7
C10—C9—H9B	109.7	C28—C27—H27B	109.7
C9—C10—H10A	109.2	C27—C28—H28A	109.4
C9—C10—H10B	109.2	C27—C28—H28B	109.4
H10A—C10—H10B	107.9	H28A—C28—H28B	108.0
C11—C10—C9	111.95 (16)	C29—C28—C27	111.15 (13)
C11—C10—H10A	109.2	C29—C28—H28A	109.4
C11—C10—H10B	109.2	C29—C28—H28B	109.4
C10—C11—H11A	109.2	C28—C29—H29A	109.4
C10—C11—H11B	109.2	C28—C29—H29B	109.4
C10—C11—C12	112.11 (16)	C28—C29—C30	111.18 (13)
H11A—C11—H11B	107.9	H29A—C29—H29B	108.0
C12—C11—H11A	109.2	C30—C29—H29A	109.4
C12—C11—H11B	109.2	C30—C29—H29B	109.4
C11—C12—H12A	109.3	C29—C30—H30A	109.3
C11—C12—H12B	109.3	C29—C30—H30B	109.3
C11—C12—C13	111.82 (17)	C29—C30—C31	111.80 (13)
H12A—C12—H12B	107.9	H30A—C30—H30B	107.9
C13—C12—H12A	109.3	C31—C30—H30A	109.3
C13—C12—H12B	109.3	C31—C30—H30B	109.3
C8—C13—C12	110.27 (17)	C26—C31—C30	109.30 (12)
C8—C13—H13A	109.6	C26—C31—H31A	109.8
C8—C13—H13B	109.6	C26—C31—H31B	109.8
C12—C13—H13A	109.6	C30—C31—H31A	109.8
C12—C13—H13B	109.6	C30—C31—H31B	109.8
H13A—C13—H13B	108.1	H31A—C31—H31B	108.3
N2—C14—H14	106.5	C2S—C1S—H1S	120.0
N2—C14—C15	111.86 (14)	C6S—C1S—H1S	120.0
N2—C14—C19	111.73 (14)	C6S—C1S—C2S	120.1 (2)
C15—C14—H14	106.5	C1S—C2S—H2S	120.1
C15—C14—C19	113.31 (14)	C3S—C2S—C1S	119.8 (2)
C19—C14—H14	106.5	C3S—C2S—H2S	120.1
C14—C15—H15A	109.5	C2S—C3S—H3S	120.3
C14—C15—H15B	109.5	C2S—C3S—C4S	119.5 (2)
C14—C15—C16	110.85 (17)	C4S—C3S—H3S	120.3
H15A—C15—H15B	108.1	C3S—C4S—H4S	119.8
C16—C15—H15A	109.5	C5S—C4S—C3S	120.4 (2)
C16—C15—H15B	109.5	C5S—C4S—H4S	119.8
C15—C16—H16A	109.3	C4S—C5S—H5S	119.9
C15—C16—H16B	109.3	C6S—C5S—C4S	120.1 (2)
H16A—C16—H16B	108.0	C6S—C5S—H5S	119.9
C17—C16—C15	111.41 (16)	C1S—C6S—H6S	120.0
C17—C16—H16A	109.3	C5S—C6S—C1S	120.05 (19)
C17—C16—H16B	109.3	C5S—C6S—H6S	120.0
			
N1—C1—C2—N2	4.0 (5)	C11—C12—C13—C8	−55.7 (3)
N1—C1—C2—C3	−177.3 (3)	C13—C8—C9—C10	−58.7 (2)
N1—C1—C3—N3	−2.2 (5)	C14—N2—C2—C1	−173.4 (2)
N1—C1—C3—C2	177.5 (3)	C14—N2—C2—C3	9.2 (4)
N1—C4—C5—C6	−175.67 (16)	C14—N2—C8—C9	68.4 (2)
N2—C2—C3—N3	−2.0 (6)	C14—N2—C8—C13	−58.1 (2)
N2—C2—C3—C1	178.3 (4)	C14—C15—C16—C17	−52.9 (2)
N2—C8—C9—C10	173.80 (16)	C15—C14—C19—C18	−53.5 (2)
N2—C8—C13—C12	−172.96 (18)	C15—C16—C17—C18	55.6 (2)
N2—C14—C15—C16	179.90 (14)	C16—C17—C18—C19	−56.5 (2)
N2—C14—C19—C18	179.06 (14)	C17—C18—C19—C14	54.6 (2)
N3—C20—C21—C22	−179.07 (13)	C19—C14—C15—C16	52.51 (19)
N3—C20—C25—C24	178.39 (12)	C20—N3—C3—C1	175.9 (2)
N3—C26—C27—C28	−174.43 (12)	C20—N3—C3—C2	−3.7 (4)
N3—C26—C31—C30	174.66 (12)	C20—N3—C26—C27	117.46 (14)
C1—N1—C4—C5	−73.7 (2)	C20—N3—C26—C31	−116.15 (14)
C1—C2—C3—N3	179.7 (3)	C20—C21—C22—C23	−55.36 (18)
C2—N2—C8—C9	−100.70 (18)	C21—C20—C25—C24	−56.42 (16)
C2—N2—C8—C13	132.84 (18)	C21—C22—C23—C24	56.07 (19)
C2—N2—C14—C15	−67.2 (2)	C22—C23—C24—C25	−56.65 (19)
C2—N2—C14—C19	61.1 (2)	C23—C24—C25—C20	56.72 (17)
C2—C1—C3—N3	−179.8 (3)	C25—C20—C21—C22	55.63 (18)
C3—N3—C20—C21	109.43 (15)	C26—N3—C3—C1	−9.3 (3)
C3—N3—C20—C25	−125.54 (14)	C26—N3—C3—C2	171.2 (3)
C3—N3—C26—C27	−57.58 (17)	C26—N3—C20—C21	−65.46 (17)
C3—N3—C26—C31	68.81 (17)	C26—N3—C20—C25	59.57 (16)
C3—C1—C2—N2	−178.7 (3)	C26—C27—C28—C29	−56.36 (17)
C4—N1—C1—C2	−15.3 (4)	C27—C26—C31—C30	−58.31 (16)
C4—N1—C1—C3	169.1 (2)	C27—C28—C29—C30	54.94 (18)
C4—C5—C6—C7	−177.2 (6)	C28—C29—C30—C31	−55.23 (18)
C4—C5—C6—C7A	170.9 (10)	C29—C30—C31—C26	56.37 (17)
C8—N2—C2—C1	−4.6 (3)	C31—C26—C27—C28	58.71 (16)
C8—N2—C2—C3	178.0 (3)	C1S—C2S—C3S—C4S	0.5 (3)
C8—N2—C14—C15	124.29 (15)	C2S—C1S—C6S—C5S	−0.6 (3)
C8—N2—C14—C19	−107.48 (16)	C2S—C3S—C4S—C5S	−0.2 (3)
C8—C9—C10—C11	55.2 (2)	C3S—C4S—C5S—C6S	−0.6 (3)
C9—C8—C13—C12	59.2 (3)	C4S—C5S—C6S—C1S	1.0 (3)
C9—C10—C11—C12	−52.4 (3)	C6S—C1S—C2S—C3S	−0.2 (3)
C10—C11—C12—C13	52.5 (3)		
